# Transfer of passive immunity and survival in Jersey heifer calves fed heat-treated pooled colostrum

**DOI:** 10.3389/fvets.2023.1094272

**Published:** 2023-02-24

**Authors:** Katherine S. Bandlow, Ailbhe King, Kelsie C. Kennicutt, Shoshana Brody, Munashe Chigerwe

**Affiliations:** ^1^Department of Veterinary Medicine and Epidemiology, University of California, Davis, Davis, CA, United States; ^2^Center for Integrative Mammalian Research, Department of Clinical Sciences, Ross University School of Veterinary Medicine, Basseterre, Saint Kitts and Nevis; ^3^Department of Population Health and Pathobiology, College of Veterinary Medicine, North Carolina State University, Raleigh, NC, United States

**Keywords:** calf, pooling, colostrum, immunity, failure of transfer of passive immunity

## Abstract

Acquisition of adequate transfer of passive immunity (ATPI) by calves depends on the absorption of sufficient mass of colostral immunoglobulin G (IgG). Several studies report conflicting evidence regarding the ability of feeding pooled colostrum to achieve ATPI. Pooling colostrum is practical and efficient for some dairies, and recommendations are required to prevent failure of transfer of passive immunity (FTPI) in calves following pooling. This study aimed to determine the effect of pooling colostrum on serum IgG concentrations, FTPI, and preweaning mortality in calves. A prospective study was performed on two conventional Jersey dairy farms where heat treatment of colostrum occurred in the same colostrum processing kitchen. Four to 10 cows contributed to colostrum pools. A sample of the colostrum pool fed to the calves and serum from calves at 24–72 h was collected for IgG concentration determination by single radial immunodiffusion assay. Multivariable and logistic regression analyses were performed to evaluate factors that predicated serum IgG concentrations and the probability of FTPI, respectively. A Cox proportional hazard model analysis was performed to determine risk factors for mortality over the preweaning period. A total of 164 calves fed 28 colostrum pools were enrolled. Birth weight, number of colostrum feedings and pool IgG concentrations were significant predictors of calf serum IgG concentrations at 24–72 h, whereas the number of colostrum feedings and age at bleeding to determine passive transfer status were not significant predictors of calf serum IgG concentrations at 24–72 h. The prevalence of FTPI was 4.9%. Birth weight, pool IgG concentrations, number of colostrum feedings, and age at bleeding to check for passive transfer status were not significant predictors of the probability of FTPI at 24–72 h. The incidence of mortality was 4.3%. Passive transfer status was not a predictor of mortality. Our study demonstrates the effect of pooling colostrum on serum IgG concentrations even in herds where colostrum with higher median colostrum IgG concentrations is fed to calves. The results emphasize the recommendations to assess pooled colostrum IgG concentrations before feeding calves.

## Introduction

Acquisition of adequate transfer of passive immunity (ATPI) by calves depends on the absorption of sufficient mass of colostral immunoglobulin G (IgG) (1). Factors affecting passive immunity include colostral IgG concentration, calf age, volume fed, colostrum collection timing, and colostrum pooling (1, 2). Calves that experience failure of transfer of passive immunity (FPTI) have higher odds for increased preweaning morbidity (3), mortality (4), increased duration of disease (5), increased pathogen shedding (6, 7), and reduced growth (8). Pooling of colostrum involves mixing of colostrum from several cows. Pooling colostrum has benefits and is still a common practice in over 50% of large US dairies (9). Pooling colostrum allows efficient heat treatment, makes colostrum immediately available to the newborn calf, and minimizes the effects of a shortage of colostrum from individual cows on dairy farms. However, pooling might decrease the IgG concentration fed to the calf due to a dilution effect. Cows producing low IgG colostrum are more likely to have larger volumes of colostrum (2). Therefore, pooling might increase the likelihood of FTPI in calves on farms where a higher number of cows with low colostral IgG concentration contribute to each colostrum pool (10). In a US national study on colostrum quality among dairies, only 25.6% of pooled colostrum samples met industry standards for IgG concentration and bacterial total plate count compared to 41.2% of non-pooled samples (11). Furthermore, the odds for FTPI were more than twice as likely for calves fed pooled colostrum compared to colostrum from individual cows (12, 13).

Pooling colostrum is practical and efficient for some dairies, and recommendations are required to prevent FTPI in calves following pooling. Several studies reported conflicting evidence regarding the ability of pooled colostrum to achieve ATPI (13–15). The studies that reported an insignificant pooling effect (14, 15) were performed when other variables affecting FTPI were controlled or eliminated. Therefore, studies, where the impact of colostrum pooling is investigated in the presence of other factors that affect FTPI, are warranted. This study aimed to determine the effect of pooling colostrum on serum IgG concentrations, FTPI, and preweaning mortality in calves. We hypothesized that pooling colostrum will increase the risk of FTPI in calves.

## Materials and methods

### Farms of study

A prospective cohort study was performed on two conventional Jersey dairy farms in Merced County, California. The two farms were within 1 km of each other and were managed by the same personnel. Both farms milked cows three times a day, at 5 am,12 pm, and 5 pm. Farm A milked 1,800 cows, whereas Farm B milked 1,300 cows. The two farms had separate milking parlors, but they processed colostrum from both farms in a single colostrum processing kitchen. Colostrum from the single kitchen was fed to calves born from both farms. Calves from both farms were housed in hutches in the same physical area.

### Colostrum processing

A colostrum pool was mixed in a custom-made 80-liter capacity pasteurizer tank before heat treatment. Colostrum was heated in the pasteurizer tank with continuous agitation until a temperature of 60°C was reached over approximately a 1-h period. The colostrum was then collected after cooling to 27°C. A 50 mL colostrum sample was collected before and after heat treatment and subsequently evaluated for IgG concentration. Colostrum was then frozen at −20°C until fed to calves. Only colostrum frozen for 24–72 h was fed to calves, whereas colostrum frozen for more than 72 h was discarded or added to milk fed to calves older than 24 h. Before feeding calves, colostrum was thawed in a water bath over 20–30 min.

### Calves

The sample size was calculated based on the national prevalence of 19.2% of FTPI in dairy calves (13), compared to a historical FTPI prevalence of 8–10% on the two farms, a type 1 error of 5%, power of 80%, and a 2-sided test using the exact Agresti-Coull method. The minimal sample size required was 132 calves. To account for a 10% drop-out due to missing values or samples, at least 145 calves were enrolled. A statistical software was used for sample size calculation (JMP Pro v16.2.0, SAS Institute, Cary, NC).

Calves were separated from cows after parturition and identified using radiofrequency ear tags and weighed using an electronic scale (WW-Paul Scales Model IQ+710, Duncan, OK). Calves were then housed in individual calf hutches. The volume of pooled colostrum fed to calves was based on the time of the day when the calves were born. Calves born between 5 am and 5 pm were fed 4 L of colostrum once by oroesophageal tubing. Calves born between 5 pm and 5 am were fed 2 L of colostrum immediately by oroesophageal tubing, then another 2 L by oroesophageal tubing at 5 am on the next day. This colostrum feeding protocol was part of the farms' standard operating procedures based on the availability of labor, and we had no influence on the protocol. After 24 h of age, calves were fed 2 L of heat-treated milk with added milk replacer three times daily, a commercial starter feed, and water, *ad libitum*. Blood was collected at 24–72 h of age, and serum was harvested after centrifugation at 2,800 × *g*. Morbidity and mortality events were recorded using an electronic record system. Calves were weaned at 10 weeks of age.

### Sample analysis

Colostrum and serum samples were analyzed for IgG concentrations using a single radial immunodiffusion assay (Triple J Farms, Bellingham, WA) according to the manufacturer's recommendations and as previously described (15). The radial immunodiffusion assay was considered the reference method for determining the colostral and serum IgG concentrations. Assessment using a Brix refractometer (Digital Brix Refractometer MA871, Milwaukee Instruments Inc, Rocky Mount, NC) was performed on colostrum and serum before freezing. Total serum solids estimation (STS) determination and Brix measurements was performed on serum using an optical refractometer (Master refractometer, Atago USA Inc, Bellevue, WA) and a Brix refractometer (Digital Brix Refractometer MA871, Milwaukee Instruments Inc, Rocky Mount, NC), respectively, according to the manufacturer's recommendations.

### Statistical analysis

Data were checked for normality using the Shapiro-Wilk test. Mean ± SEM were reported when data were normally distributed, whereas median and range were reported when data were not normally distributed. Descriptive statistics for calves included the number of calves enrolled, birth weight, volume and frequency of colostrum administered in the first 24 h of life, total serum solids (Brix and optic refractometry), serum IgG concentrations at 24–72 h, apparent efficiency of absorption (AEA), proportions of calves with failure of transfer of passive immunity (FPTI), average daily gain (ADG), and mortality events. FTPI was defined as those calves with serum IgG of ≤20 g/L (16). The apparent efficiency of absorption was calculated as previously described (17).

Association between the number of colostrum feedings (1 feeding of 4 L or 2 feedings of 2 L within 24 h, which was dictated by the time of birth), age of calves at blood collection for serum IgG determination (24, 48, or 72 h) pool identification (day and time of colostrum collection), and AEA were determined using multivariable linear mixed model regression analysis. The number of colostrum feedings and age of calves at the time of feeding colostrum were considered fixed effects, whereas pool identification was considered a random effect. Birth weight and colostral IgG concentrations were not considered in the multivariable linear mixed model regression analysis to predict AEA because these parameters are included in the calculation of AEA. Similarly, the total colostrum volume fed to the calves was not considered a predictor of AEA because all calves were fed 4 L within 24 h.

A multivariable linear mixed model regression analysis was performed to evaluate factors predicting serum IgG concentrations as a continuous variable in calves at 24–72 h. In the model, birth weight, pool IgG concentrations, age at the time of assessing passive transfer status, and number of colostrum feedings (4 L once or 2 L twice in 12 h), were considered fixed effects, whereas pool identification was considered a random effect. A multivariable mixed model logistic regression analysis predicting the probability of FTPI (yes or no) was determined with birth weight, pool IgG concentrations, age at the time of assessing passive transfer status, and number of colostrum feedings (4 L once or 2 L twice in 12 h) considered as predictor variables.

Multivariable linear mixed model regression analysis was performed to evaluate factors that predicted ADG with serum IgG concentration at 24–72 h, number of colostrum feedings, and age of calf at the time of passive immunity status check considered fixed effects, whereas pool identification was considered a random effect. Calves were weighed at weaning using an electronic scale (WW-Paul Scales Model IQ+710, Duncan, OK). Average daily gain was calculated by dividing the difference between the birth weight and weaning weight and the age at weaning (70 days). The ADG for calves that died was calculated by dividing the difference between the birth weight and the weight on the day the calf died by the calf's age in days at death.

In the linear and logistic regression analysis, model building was first performed by univariable analysis, followed by the inclusion of variables with *P* < 0.2 in the multivariable analysis. In the multivariable linear regression models, multicollinearity among the variables was assessed using the coefficient of determination (R^2^) for each variable with other variables and the variance inflation factor [1/(1-R^2^)]. Values of *R*^2^ > 0.75 and variable inflation factors >4 indicated multicollinearity (18). Model fit assessment for the multivariable linear regression analysis was assessed by the overall *F*-test value, normality of the residuals, and magnitude of the coefficient of multiple determination (*R*^2^). Model fit assessment for the logistic regression model was assessed using log-likelihood estimation.

Due to the low number of mortality events on the farms, a univariable exact logistic regression was performed with serum IgG concentration as the explanatory variable and mortality as the outcome variable. A Cox proportional hazard model and subsequent determination of risk ratios with 95% confidence intervals (95% CI) for pre-weaning mortality as a function of calf serum IgG concentrations at 24–72 h was performed for the preweaning period. Hazard ratios >1 or <1, excluding 1, and a corresponding *P* < 0.05 were considered significant. Calves with serum IgG concentrations ≤20 g/L were considered to have FTPI (16). In all analyses, JMP Pro v16.2.0 and SAS v9.4 (SAS Institute, Cary, NC) and Graph Pad Prism v9.4.0 (Graph Pad Software, La Jolla, CA). *P* < 0.05 was considered significant.

## Results

### Colostrum pools

Two-hundred-and-two cows were enrolled and contributed to 28 pools of colostrum. The number of cows contributing to each pool ranged from 4 to 10. The median (range) pool volume was 55.6 L (26.6–70.7). The median (range) individual colostral IgG concentration for all cows was 74.4 g/L (1.8–131.7). The median (range) colostral Brix refractometer measurement for individual cows was 22.8 % (10–31.5). Median (range) pool colostral IgG concentrations before and after heat treatment were 84.7 (44.7–126.8) and 70.4 (40.7–106.7) g/L, respectively. The median decrease in pool colostral IgG concentrations after heat treatment was 9 g/L (*P* < 0.0001). The median (range) pool colostral Brix refractometer measurement before and after heat treatment was 22.9 % (20.5– 25.2) and 22.3 % (20.5–25.4), respectively. There was no significant difference (*P* = 0.092) between the Brix refractometer measurements before and after heat treatment. Summary table of colostrum pools is available in Supplementary material.

### Calves

Zero calves were fed colostrum from two pools (pool 4 and 15) and were excluded from analysis. A total of 164 calves fed colostrum from the remaining 26 pools were enrolled. The median (range) number of calves fed each colostrum was 6 (1–12). The mean ± SD birth weight was 25 ± 3.4 kg. Fifty-six calves were fed 4 L of colostrum once within the first 12 h after birth, whereas 108 calves were fed 2 L of colostrum twice within the first 12 h after birth. The range of age for when the calves were fed the first colostrum meal was 2–7 h. The median (range) AEA of IgG for all calves was 20.6% (7.9–56.5). Calves fed 2 L of colostrum twice within 12 h had a higher AEA compared to calves fed 4 L once within 12 h (24.7 *vs*. 19.6; *P* = 0.038). The age of calves at the time of passive transfer status check was not a predictor of AEA (*P* = 0.156). The median (range) for STS and serum Brix measurements was 6.4 g/dL (4.8–8.7) and 9.8% (6.9–12.2), respectively.

Median (range) serum IgG concentrations were 34.7 g/L (11.0–55.2). The distribution of serum IgG concentrations is summarized in Table 1. Proportions of calves with FTPI were 4.9% (8/164). Birth weight (*P* < 0.0016) was negatively associated with calf serum IgG concentrations at 24–72 h. In contrast, pool IgG concentrations (*P* < 0.0001), and the number of colostrum feedings (*P* = 0.002) were positively associated with calf serum IgG concentrations at 24–72 h. The age at bleeding to check for passive transfer status (*P* = 0.819) was not a significant predictor of calf serum IgG concentrations at 24–72 h. The multivariable linear regression analysis model is summarized in Table 2. In the logistic regression, birth weight (*P* = 0.176), pool IgG concentrations (*P* = 0.551), number of colostrum feedings (*P* = 0.160), and age at bleeding to check for passive transfer status (*P* = 0.998) were not significant predictors of probability of FTPI at 24–72 h.

**Table 1:** Distribution of serum IgG concentrations in 164 Jersey calves assessed for passive transfer status at 24–72 h.

**Serum IgG concentrations range (g/L)**	***N* (%)**	**TPI status**
<10.0	0 (0)	Poor
10.0–17.9	7 (4.3)	Fair
18.0–24.9	29 (17.7)	Good
≥25.0	128 (78.0)	Excellent

Transfer of passive immunity status based on the consensus statement by Lombard et al. (19).

**Table 2:** Summary of multivariable linear regression model predicting calf serum IgG concentrations as a continuous variable at 24–72 h in 164 Jersey calves fed various pools of colostrum.

**Variable**	**Estimate (95% CI)**	***P*-value**	**^†^VIF**	**^†^*R*^2^ with other variables**
Intercept	6.12 (−3.13, 15.42)	0.193	-	-
Birth weight	−0.78 (−1.26, −0.30)	<0.0016	1.04	0.04
Pool IgG concentration	0.42 (0.38, 0.47)	<0.0001	1.08	0.07
Number of colostrum feedings (2 feedings)	5.26 (1.91, 8.62)	0.002	1.03	0.03
Age at bleeding to check for passive transfer status	−0.60 (−5.77, 4.57)	0.819	1.08	0.07

Colostrum pool identification, that is, day and time of collection of colostrum (random effect) was not associated with serum IgG concentrations at 24–72 h of age.

Adjusted R^2^ for the model = 0.87.

VIF, variance inflation factor; 95% CI, 95% confidence interval; R^2^, coefficient of multiple determination.

^†^Values of R^2^ > 0.75 and variable inflation factors >4 indicated multicollinearity (18).

Median (range) for ADG was 0.59 kg (0.39–0.77). Serum IgG concentration at 24–72 h positively predicted ADG (*P* = 0.031). In contrast, the frequency of feeding colostrum (*P* = 0.737), and the age of the calf at the time of passive immunity status check (*P* = 0.092), were not significant predictors of ADG. Pool identification (random effect) was not a significant variable (*P* > 0.05) when it was included in the models predicting serum IgG concentrations at 24–72 h, FTPI, or ADG.

Although the farms had detailed standard operating procedures for managing sick calves, observation by the investigators indicated that the diagnosis of specific morbidity events and treatments was inconsistent among the calf caretakers. Therefore, the analysis of morbidity events was excluded from the analysis. Seven calves died (4.3%) secondary to diarrhea before weaning. The age range for when the calves died was 2–43 days. Serum IgG concentration was not a significant (*P* = 0.668) predictor variable of mortality in the univariate logistic regression model. Serum IgG concentration was not a significant predictor of mortality during the preweaning period (hazard ratio = 1.08; 95% CI, 0.52, 2.23; *P* = 0.835).

## Discussion

Colostrum pool IgG concentrations, and birth weight were predictors of calf serum IgG concentrations at 24–72 h. The effect of pool colostrum IgG concentrations on calf serum IgG concentrations contrasts with previous studies that reported an insignificant effect of pooling on calf serum IgG (14, 15). The difference between our studies might be that in the previous studies, other variables affecting serum IgG concentrations were controlled or eliminated. Additionally, the number of cows contributing to each pool was fewer (four contributing to a collection) and maintained constant (15). In our study, the number of cows contributing to a pool ranged from 4 to 10. It should be noted that birth weight, pool IgG concentrations, and number of colostrum feedings were not significant predictors of the probability (logistic regression) of FTPI at 24–72 h in contrast to when serum IgG concentrations were determined as a continuous variable. This is most likely due to our study's low frequency of FTPI (4.9%). The proportions of calves with various serum IgG concentrations were considered acceptable based on the most recent recommendations for dairy herds (19).

The calf's birth weight was negatively associated with serum IgG concentrations at 24–72 h. The association between birth weight and serum IgG at 24–72 h could not be interpreted further because all calves were fed a similar volume of colostrum.

The median AEA reported in our study was consistent with previous studies (17, 20). The AEA for calves fed colostrum over two feedings was higher than when calves were fed once. Absorption of colostrum components is a competitive process, therefore larger volumes fed at once may decrease absorption of colostrum of IgG because of mechanical distension of the abomasum and other forestomachs, leading to a reduction in abomasal emptying into the small intestine where absorption of colostral components occur (21). The study farms protocols for feeding colostrum once or twice depending on the time the calves were born were based on the availability of labor. The number of feedings (4 L once or 2 L twice) was associated with serum IgG concentrations at 24–72 h in our study, consistent with the aforementioned difference in AEA and with previous studies that reported that calves administered a second colostrum feeding had lower odds (odds ratio <1) of FTPI, a lower probability of being treated for respiratory disease, diarrhea, or any disease, and a greater pre-weaning ADG (22). In the study by Abuelo and others (22), calves fed once only received 3 liters of colostrum, whereas calves fed a second feeding received a total of 4 liters.

Serum IgG concentrations at 24–72 h were positively associated with ADG. This result might reflect that calves with an adequate transfer of passive immunity are unlikely to experience severe comorbidities that significantly affect their appetite (23). The prevalence of preweaning mortality reported in our study (4.3%) was lower than the reported national average of 5% (24). Serum IgG concentration at 24–72 h was not a predictor of mortality during the preweaning period. However, this might be due to the low mortality rate reported in our study.

The practical implications of our study results demonstrate that colostrum pool IgG concentrations affect serum IgG concentrations, including farms that feed colostrum with high median colostrum IgG concentrations as in our study. Therefore, recommendations to assess colostrum from individual cows with instruments such as the Brix refractometer before feeding applies to pooled colostrum. Farms that achieve higher serum IgG concentrations in calves at 24–72 h, as in our study have a lower prevalence of preweaning mortality (1).

The limitations of our study include a lack of recorded consistent morbidity events for the calves. Availability of morbidity events would have allowed determination of the association between serum IgG concentrations and morbidity. The number of calves classified as FTPI and the number of calves which died were low which may have affected the power of our statistical tests and determination of associations between explanatory variables and the outcomes. Our study is from a single geographical region using a single breed and has limited external validity. The results of our research apply to farms with Jersey cattle that heat treat colostrum and feed pooled colostrum. Although this reflects routine practices on a dairy farm, the number of calves fed from each pool varied. Therefore, the sample size of calves enrolled in our study was affected by pool volume and the number of calves born.

## Conclusions

Pooling colostrum, number of colostrum feedings and birth weight affect serum IgG concentrations at 24–72 h in calves fed a constant volume of colostrum. However, when the prevalence of FPTI and mortality is low, pool IgG concentrations do not predict the probability of FPTI or mortality. Pooled colostrum should be assessed with instruments such as the Brix refractometer before feeding. Our study demonstrates the effect of pooling colostrum on serum IgG concentrations even in herds where colostrum with higher median colostrum IgG concentrations is fed to calves.

## Data availability statement

The original contributions presented in the study are included in the article/Supplementary material, further inquiries can be directed to the corresponding author.

## Ethics statement

The animal study was reviewed and approved by University of California Davis Institutional Animal Care and Use Committee (#22251). Written informed consent was obtained from the owners for the participation of their animals in this study.

## Author contributions

KB, AK, KK, SB, and MC: investigation, methodology, formal analysis, writing, and editing. KB, AK, and MC: funding acquisition and project administration. AK and MC: conceptualization and supervision. KB and MC: visualization and writing—original draft. MC: resources. All authors contributed to the manuscript and approved the submitted version.
