# Supplementary material for: Transfer of passive immunity and survival in Jersey heifer calves fed heat-treated pooled colostrum
Source: Front Vet Sci. 2023 Feb 24;10:1094272. doi: 10.3389/fvets.2023.1094272 (PMC9998901; doi:10.3389/fvets.2023.1094272)
Supplement: Supplementary file 1 [file Table_1.docx]

**Supplementary Table 1:** Summary table of colostrum pools.

^*^Zero calves were fed colostrum pools 4 and 15 and were excluded from analysis.

| **Pool ID** | **Total number of cows contributing to pool** | **Total number of calves fed from pool** | **Total volume (L) of pooled colostrum** | **Pooled IgG concentration after heat treatment (Brix %)** | **Pooled IgG concentration after heat treatment (RID g/L)** |
| --- | --- | --- | --- | --- | --- |
| 1 | 5 | 2 | 57.2 | 23.4 | 69.64 |
| 2 | 9 | 1 | 56.7 | 25.2 | 62.04 |
| 3 | 8 | 10 | 26.6 | 24.4 | 68.7 |
| *4 | 5 | 0 | 36.1 | 23 | 62.48 |
| 5 | 5 | 2 | 37.0 | 22.3 | 80.4 |
| 6 | 8 | 10 | 69.4 | 21.9 | 78.4 |
| 7 | 8 | 8 | 70.7 | 21.5 | 55.64 |
| 8 | 8 | 4 | 47.4 | 21.9 | 73.76 |
| 9 | 5 | 6 | 37.0 | 22.3 | 73.6 |
| 10 | 9 | 8 | 61.2 | 22.2 | 102.4 |
| 11 | 6 | 10 | 37.5 | 25.4 | 95.88 |
| 12 | 8 | 12 | 59.6 | 23.1 | 66.44 |
| 13 | 9 | 5 | 51.5 | 21.2 | 65.72 |
| 14 | 8 | 7 | 60.9 | 21.7 | 56.08 |
| *15 | 4 | 0 | 29.5 | 20.4 | 63.16 |
| 16 | 9 | 6 | 61.0 | 24.5 | 59.36 |
| 17 | 9 | 6 | 68.2 | 22.2 | 73.04 |
| 18 | 6 | 7 | 37.6 | 21.6 | 62.28 |
| 19 | 6 | 2 | 54.9 | 24.4 | 106.7 |
| 20 | 10 | 9 | 66.1 | 22.2 | 40.45 |
| 21 | 8 | 11 | 51.5 | 24.5 | 48.64 |
| 22 | 8 | 5 | 64.4 | 22.6 | 79.85 |
| 23 | 8 | 5 | 55.6 | 21.8 | 99.4 |
| 24 | 9 | 6 | 66.1 | 21.1 | 42.6 |
| 25 | 6 | 5 | 40.7 | 22.2 | 85.96 |
| 26 | 5 | 4 | 43.1 | 23.2 | 71.15 |
| 27 | 8 | 7 | 51.6 | 22.9 | 97.4 |
| 28 | 4 | 6 | 35.5 | 24.9 | 101.16 |
|  |  |  |  |  |  |
|  |  |  |  |  |  |
